# Basal Metabolic Rate, Rheumatoid Arthritis and Alzheimer’s Disease: Insights from a genetic correlation and Mendelian randomization study

**DOI:** 10.1002/alz70861_108452

**Published:** 2025-12-23

**Authors:** Yunyun Guo

**Affiliations:** ^1^ The Ageing Epidemiology (AGE) Research Unit, School of Public Health, Imperial College London, London UK

## Abstract

**Background:**

Previous studies, including Mendelian randomisation (MR), have suggested an association between metabolism and inflammatory disease risk and Alzheimer's disease (AD) risk, respectively. However, few studies have explored the link between the three, especially the potential pathways of metabolic effects on AD risk, such as the role of rheumatoid arthritis (RA) and its blood markers.

**Method:**

We performed 2‐sample MR analyses of basal metabolic rate (BMR) and AD. MR analyses were performed using an inverse variance weighting method, supplemented by a series of sensitivity analyses. Considering the close relationship between RA, RA‐associated blood markers, RA treatment, and AD, we applied 2‐step MR to assess whether the identified effects were mediated by modulation of RA, RA‐associated blood markers, and methotrexate.

**Result:**

Our univariate MR analyses showed for each standard deviation increase in relative BMR, the risk of AD was reduced by 22% (OR = 0.78, 95% CI: 0.69 to 0.88, *p* < 0.001). The effect of BMR on AD remained after accounting for height, weight, body mass index (BMI), waist‐to‐hip ratio and body fat percentage. Elevated BMR resulted in an increased risk of RA (OR = 1.24, 95% CI: 1.06 to 1.44, *p* = 0.006), especially seropositive RA (OR = 1.29, 95% CI: 1.09 to 1.53; *p* = 0.003) as well as an increased methotrexate use (OR = 1.0013, 95% CI: 1.0001 to 1.0026, *p* = 0.032). RA was not associated with future AD risk after accounting for the role of BMR, but the results suggested that methotrexate use was a protective factor for future AD risk (OR = 2.92e‐05; 95% CI: 1.97e‐08 to 4.35e‐02, *p* = 0.005). We also performed mediation analyses to estimate potential mediators and showed that the effect of BMR on AD risk was not mediated by RA, seropositive RA, seronegative RA, C‐reactive protein (CRP), erythrocyte sedimentation rate (ESR), interleukin‐1β (IL‐1β), tumor factor necrosis‐α (TNF‐α), matrix metallopeptidase 3 (MMP3) and methotrexate.

**Conclusion:**

Our study reveals that BMR has a protective causal effect on AD. Mediation analyses by two‐step MR showed that this effect was not mediated by rheumatoid arthritis (RA), RA‐associated blood markers, and methotrexate use.

